# Expression of Molecular Markers Associated with Tenosynovial Giant Cell Tumours and Bone Destruction: A Systematic Review

**DOI:** 10.3390/jcm15062238

**Published:** 2026-03-15

**Authors:** Thomas R. W. Ward, Feier Zeng, Robert U. Ashford, Nicholas C. Eastley, Ning Wang

**Affiliations:** 1 Orthopaedic Department, University Hospitals of Leicester NHS Trust, Leicester LE1 5WW, UK; 2 Leicester Cancer Research Centre, School of Medical Sciences, University of Leicester, Leicester LE1 5WW, UK

**Keywords:** Tenosynovial Giant Cell Tumour (TGCT), Pigmented Villonodular Synovitis (PVNS), osteoclast, bone destruction, osteolysis, CSF-1

## Abstract

**Background/Objectives**: Tenosynovial giant cell tumours (TGCT) are a group of mesenchymal tumours involving the synovium, bursae, and tendon sheaths, comprising two subtypes: nodular and diffuse. Although predominantly benign, diffuse forms can be locally aggressive, resulting in bone destruction. The pathogenesis of TGCTs is still poorly understood. The aim of this study was to systematically review the current literature on the factors, mechanisms, and markers involved in TGCT disease, focussing on their potential role in bone destruction. **Methods**: This systematic review was conducted using the PRISMA guidelines. A search was performed using PubMed, Scopus, and Cochrane Library, and all original scientific research into mechanisms/pathways/signalling involving TGCTs was included. **Results**: After the review process, 51 studies were included for data extraction. Extracted data included authorship, publication year, patient numbers and aetiology (nTGCT/dTGCT), demographics, investigative methods, and studied biological factors, mechanisms, and markers. Cross-tabulation of reported elements revealed 159 unique factors, with most appearing only once. Eight elements were reported five or more times: CSF1, CD68, Ki-67, MMP9, CD163, TRAP, TNF-α, and IL-1β. Although representing just 5% of all identified factors, these appeared in 69% of the included studies, highlighting their prominence in the literature. **Conclusions**: Apart from the well-known osteoclastogenesis factor CSF1, inflammatory cytokines (TNF-α and IL-1β) and monocyte–macrophage lineage makers (CD68, CD163) are signalling pathways key to TGCT disease progression and associated bone destruction.

## 1. Introduction

Tenosynovial giant cell tumours (TGCTs) are a group of rare mesenchymal tumours that arise from the joint synovium, bursae, and tendon sheaths, and may be intra- or extra-articular. They were first described in 1852 as Pigmented Villonodular Synovitis (PVNS) by Édouard-Pierre-Marie Chassaignac due to the features of nodular damage of the synovial membranes [1,2]. In 1967, Granowitz and Mankin classified PVNS based on its growth pattern, suggesting localised disease should be seen as a firm soft-tissue mass attached to the deep structures and as a potential cause of mechanical derangement of the knee, and the diffuse PVNS is more aggressive [3]. The World Health Organisation (WHO) reclassified TGCTs as a group in 2013 to encompass PVNS and giant cell tumours of the Tendon Sheath (GCTTS) [4], although these terms are still incorrectly used in the current literature. The 2013 classification of TGCTs divided the disease spectrum into two specific subtypes: nodular and diffuse.

Nodular TGCT (nTGCT) commonly presents as a small, isolated firm lesion that is often intra-articular. In comparison, diffuse TGCT (dTGCT) is larger (often >5 cm), showing extensive involvement of the synovium, with extension into the extra-articular structures not being uncommon. Although both are classified under the TGCT disease spectrum, they present with great variation in their phenotypic expression. Although TGCT can affect any joint, nTGCT is more common in the hands (particularly in the digits [5]), whereas dTGCT tends to affect larger joints. Over 60% of all dTGCT cases are found in the knee, with a further 25% found in either the hip or ankle [6].

Diagnosis of TGCT from clinical symptoms and imaging alone is not always conclusive, and differential diagnoses should be excluded. Differential diagnoses include conditions such as rheumatoid arthritis, which typically presents with poly-arthropathy and systemic inflammatory features. Synovial chondromatosis is characterised by multiple intra-articular cartilaginous loose bodies, and haemophilic arthropathy or haemosiderosis, which may show increased hemosiderin deposition similar to TGCT, and intra-operatively carries the same brown colour change from normal synovial tissue. Although the majority of differentials are also benign diseases, it is important to exclude malignant conditions such as synovial sarcoma, a malignant neoplasm that can mimic TGCT radiologically but demonstrates aggressive features and distinct histopathology [7]. To gain an accurate diagnosis, in addition to clinical history, examination, and imaging, a biopsy may be necessary if there is any uncertainty [8].

The incidence of TGCT is greatest between the third and sixth decades of life. nTGCT is the more common subtype, with an annual incidence reported between 12 and 44/million patients [9,10]. dTGCT is rarer, with reported rates from 4 to 11/million patients. Previously, TGCT was thought to have an equal sex distribution; however, recent studies have suggested there may be a slightly greater female predilection [9,11].

Macroscopically, nTGCTs have a well-circumscribed lobulated appearance with a yellow/brown colour and are often less than 4 cm in size. dTGCTs are often larger, at least 5 cm, and appear as a yellow/brown spongy tissue that is firm and nodular, covering the synovial surface. Histologically, TGCTs are identified by the presence of mononuclear cells, multinucleated giant cells, foamy macrophages, inflammatory cells, and hemosiderin [12]. TGCTs are benign tumours, although rare malignant transformation has been reported in case reports and a small case series [13,14]. When malignant transformation occurs, it is more likely to affect the lower extremities in middle-aged to older adults. Malignant TGCT can arise *de novo*, but it is the most common after multiple recurrences of the diffuse form.

The clinical presentation of TGCT varies depending on the joint affected. Common symptoms include swelling, stiffness, a palpable lump/mass, pain that is exacerbated by movement, and mechanical symptoms. When large joints such as the knee are involved, mechanical symptoms include catching, locking, popping, giving way, and weakness [15]. In the United Kingdom (UK), despite a recent global consensus meeting, there are currently limited guidelines for the management of TGCT [8]. TGCT is investigated, managed, and followed up on with great variability across the UK [16].

Bone destruction is one of the most severe sequalae of aggressive dTGCTs and can cause significant morbidity. Aggressive dTGCTs often result in the need for multiple surgeries, and chondral/osseous destruction can result in established osteoarthritis requiring arthroplasty. The pathogenesis of bone destruction in TGCTs is poorly understood. Bone destruction is thought to be osteoclast-mediated. Osteoclasts are multinucleated cells of the monocyte/macrophage haematopoietic lineage involved in normal bone homeostasis [17]. Osteoclasts differentiate under the control of two main cytokine factors—Receptor Activator of Nuclear factor Kappa-Β (RANK) ligand and macrophage colony-stimulating factor (M-CSF) [18,19], although many other signalling factors and pathways also influence osteoclast activity and expression.

Overexpression of colony-stimulating factor 1 (CSF1) due to chromosomal translocations involving the *CSF1* gene on chromosome 1p13 has been reportedly present in the majority of TGCT cases [20]. At a molecular level, both nodular and diffuse subtypes share the presence of a fusion involving the *CSF1* gene, which drives tumour growth.

Although both subtypes share this common genotype, their phenotypes and clinical presentations vary greatly. Furthermore, variation can also be seen between cases of dTGCT, making the disease course hard to predict. Secretion of factors and cytokines like CSF1, which facilitate cancer cell proliferation and enhance tumour growth, has been proven in malignant cancers, such as lung and prostate [21]. These specific cancers have a predisposition to metastasise to bone and result in bone resorption and lysis.

Although CSF1 expression has been widely accepted as a factor involved in the development of TGCT, the involvement of many other intertwined signalling pathways means the pathogenesis of TGCT is still poorly understood [22]. The aim of this study was to systematically review the current literature and research on TGCTs, focussing on the signalling factors, molecular mechanisms, and pathways involved in disease pathogenesis.

## 2. Materials and Methods

This systematic review was conducted in accordance with the Preferred Reporting Items for Systematic Reviews and Meta-Analyses (PRISMA) guidelines. A comprehensive literature search was performed of the PubMed, Scopus, and Cochrane databases using the specific search criteria found in Appendix A. Eligible studies were identified based on the following inclusion and exclusion criteria. The PRISMA checklists can be found in the Supplementary Materials.

### 2.1. Inclusion Criteria

- Original scientific research into mechanisms/pathways/signalling involving TGCT.
- Aetiology of TGCT in the form of nTGCT or dTGCT.
- All ages.
- All date ranges (database inception to search date 12 January 2026).
- Articles in English.

### 2.2. Exclusion Criteria

- Case reports.
- Randomised clinical trials.
- Reviews of current literature.
- Interventional studies.
- Articles where the full text was not available.
- Non-English articles with no official or authenticated translation.

Titles and abstracts were screened independently by two reviewers (T.R.W.W and F.Z) for inclusion. Discrepancies were resolved through discussion with the senior author (N.W). After initial screening, potentially relevant abstracts were extracted for full-text assessment. Full texts were similarly reviewed by the same two reviewers prior to inclusion, with any conflicts again resolved by discussion with the senior author.

### 2.3. Data Extraction

Data was extracted from the included studies and collated in a formatted Excel spreadsheet, then transferred to a standardised table. The data extracted included authors, year of publication, patient numbers and demographics, tumour subtype (nTGCT/dTGCT) where available, primary investigation techniques, and main mechanisms/pathways/signalling investigated.

### 2.4. Quality Assessment

The methodological quality and risk of bias of included studies were assessed using the Mixed Methods Appraisal Tool (MMAT), developed by McGill University [23]. The MMAT was selected due to its suitability for systematic reviews incorporating heterogeneous study designs, including quantitative descriptive and quantitative non-randomised studies. This tool enables the concurrent appraisal of methodological rigour across diverse evidence types using a standardised set of design-specific criteria. Each study was evaluated against the five core criteria relevant to its methodological category, focussing on domains such as appropriateness of sampling strategy, adequacy of measurements, completeness of outcome data, risk of confounding, and coherence between qualitative data sources and interpretations. The quality and risk of bias of the included papers were assessed by two reviewers, T.R.W.W and F.Z, to enhance reliability, with discrepancies resolved through discussion and, when necessary, consultation with a third reviewer, N.W. Each domain was classified as either Yes (low risk of bias), Can’t tell (CT) (moderate risk), or No (high risk of bias).

### 2.5. Publication Bias

Formal statistical assessment of publication bias (e.g., funnel plots) was not conducted, as no meta-analysis was performed

## 3. Results

### 3.1. Search Results

Comprehensive searches of the PubMed, Scopus, and Cochrane databases were performed using the specific search criteria found in Appendix A, and they yielded 324, 615, and 33 results, respectively, providing a total of 972 articles. Using the title, DOI, and PMID where available, 245 duplicates were identified and removed, resulting in 727 articles for title and abstract review. Screening by two independent reviewers using the above criteria and resolution of conflicts resulted in the exclusion of 652 articles, leaving 75 articles for full-text review. A Cohen’s kappa value of 0.76204 was achieved, which represents substantial agreement between the two raters. After an extensive search, the full-text articles were sourced for 67 results, and 8 were unable to be retrieved and were therefore excluded [24,25,26,27,28,29,30,31].

**Figure 1:**
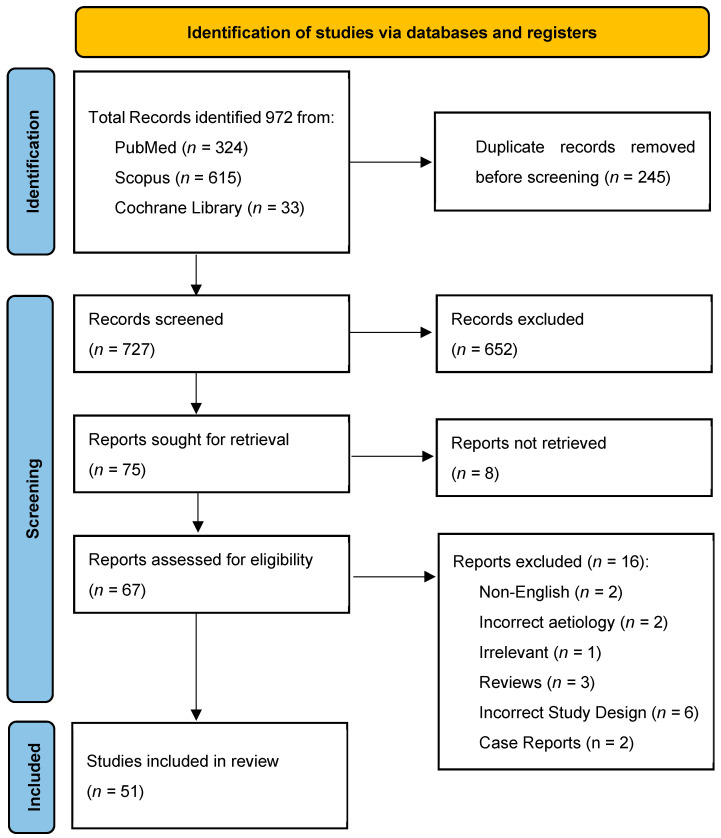
PRISMA flow chart of the inclusion process.

The 67 available full texts were again independently reviewed, and after any conflicts were resolved, 16 were excluded [32,33,34,35,36,37,38,39,40,41,42,43,44,45,46]. Reasons for exclusion were due to publication in a non-English language (*n* = 2), incorrect aetiology (*n* = 2), irrelevance (*n* = 1), reviews of evidence (*n* = 3), and incorrect study design, e.g., interventional study (*n* = 6) and case reports (*n* = 2). This resulted in 51 articles being included in the study for data extraction. A PRISMA flow chart of the inclusion and review process is shown in Figure 1. There was no date restriction on the searches from the inception of the respective databases to 12 January 2026.

### 3.2. Data Extraction

Data extracted included first author name, year of publication, patient numbers including aetiology split of nTGCT and dTGCT where available, patient characteristics including age range and average age were reported as either the mean or median, gender split for each aetiology where available, primary investigation techniques, and main factors, mechanisms, pathways, and signalling investigated. This data was collated and is shown in Table 1.

### 3.3. Quality Assessment

The risk of bias of the included studies is reported in Table 2. Studies included were either quantitative non-randomised studies or quantitative descriptive studies, with 25 and 26 studies in each category, respectively. Criterion 4.4., “Is the risk of nonresponse bias low?”, was not applicable for all but one study, as it was not pertinent for the remaining study types. The overall risk of bias for all studies included was ‘moderate’ to ‘low’, and for that reason, no study was excluded from this review solely based on its quality.

### 3.4. Analysis

Once each paper had been reviewed and the factors, mechanisms, pathways, and signalling investigated were recorded (Table 1), each individual element was cross-tabulated with each paper to identify trends and frequencies. There were 159 unique aspects identified, and on cross analysis, 112 were reported once, 27 were reported twice, five were reported three times, six were reported four times, three were reported five times, one was reported six times, one was reported eight times, one was reported nine times, one was reported eleven times, and one reported fourteen times.

A total of eight elements were reported five or more times, which included CSF1 (*n* = 14), Cluster of Differentiation 68 (CD68) (*n* = 11), Antigen Kiel 67 (Ki-67) (*n* = 9), Matrix Metalloproteinase: 9 (MMP9) (*n* = 8), Cluster of Differentiation: 163 (CD163) (*n* = 6), Tartrate-Resistant Acid Phosphatase (TRAP) (*n* = 5), Tumour Necrosis Factor alpha (TNF-α) (*n* = 5), and Interleukin-1 beta (IL-1β) (*n* = 5). These eight elements comprise only 5% of the different elements investigated, and yet were reported in 35 out of the 51 (69%) papers included in the study; the full cross-tabulation is shown in Table 3.

### 3.5. CSF1

CSF1 expression has been shown to be statistically significantly increased at the mRNA and protein level in TGCT compared to control groups [54,57,69,73], as well as other solid tumours [64]. Expression was reported to be 6.9 times greater, with expression levels of 3.51 compared to 24.22, respectively, when estimated using the ratio of fluorescence intensity compared to Glyceraldehyde 3-phosphate dehydrogenase (GAPDH) [73]. Positive staining was observed mainly in mononuclear cells and some multinuclear giant cells [79,93]. Ota et al. reported that high positivity of CSF1 was observed in 85% of cases and was significantly correlated with osteochondral lesions (*p =* 0.009). Although CSF1 and CSF1 receptor expression were increased compared to control tissue, the differences were not statistically significant when comparing with local recurrence (*p* = 1 and *p* = 0.124, respectively) [79]. A gene expression analysis showed that cells in neoplastic clusters expressed high levels of CSF1 as compared with the non-neoplastic cell types in TGCT [87]. *CSF1* gene translocations on chromosome 1 (1p13) have been commonly identified through fluorescence in situ hybridization (FISH) [67,82], with translocations involving FN1, PDPN, EBF1 [87], CD101, COL6A3, PRG4, TNC, and CDH17 [64,74].

### 3.6. CD68

The synovium of TGCT was characterised by the increased infiltration of CD68-positive macrophages compared to control tissue in studies looking at CD68 in both mononucleated and giant cells. Yoshida et al. reported that >30% of all cells under high-power fields in each section showed positive staining [93]. There was some variability in the presence of mononucleated cells, with 76% positivity in small mononuclear and foamy cells [81,83]. Immunostaining showed a positive cytoplasmic reaction in “most” of the proliferating synovial cells compared to non-proliferating cells [50]. CD68 expression was similar in nTGCT and dTGCT on double immunofluorescence and flow cytometry (*p* = 0.3871 and *p* = 0.31, respectively), although the number of double-positive CD68+/h4Ph+ was significantly lower in nTGCT compared to dTGCT (*p =* 0.015) [50].

### 3.7. Ki-67

Although studies showed a positive Ki-67 proliferative index or immunohistochemical staining in mononuclear cells localised to the cell nucleus [72], multinucleated giant cells were negative for Ki-67 [89]. There was a significant difference in activity between synovial stromal and lining cells [51]; however, there was a significantly higher result in TGCTs compared to osteoarthritic control tissue [53]. There was no statistical significance between nTGCTs and dTGCTs. Berger et al. showed moderate and high Ki-67 proliferation rates in both nTGCTs (91.3%, 11.5%) and dTGCTs (89.5%, 8.7%), but no statistical significance between the groups (*p* = 0.65) [49,90]. Although, in contrast, Weckauf et al. reported that a high proliferation rate was seen more in dTGCTs than nTGCTs, this was still not statistically significant between the groups (*p* value not reported) [49,90]. There was a reported difference between primary and recurrent cases, but disagreement between papers on whether it was statistically significant [89,90].

### 3.8. MMP9

Expression levels of MMP9 in fibroblasts were significantly higher in PVNS than in controls of osteoarthritis and rheumatoid arthritis groups, with significantly higher expression of genes related to inducing inflammatory response and bone destruction pathways (*p* ≤ 0.001) [69]. Immunohistochemical staining was diffuse and the most positive in giant cells, then in histiocytes, with fibroblasts showing the least intensity. MMP9 was mainly cytoplasmic, with small amounts in the extracellular matrix [89]. TGFβ was reported to promote the increased expression of MMP9 in TGCT compared to osteoarthritis and normal control tissue (*p* ≤ 0.001 and *p* ≤ 0.05, respectively). It has also been shown to enhance the aggressive behaviour of the fibroblasts [71]. Despite this, O’Keefe R J et al. showed there was no correlation between radiographic aggressiveness and MMP9 expression [78].

### 3.9. CD163

CD163 was shown to be increasingly positively stained on immunohistochemical analysis [52,82,88], particularly in proliferating synovial cells, and compared to control synovial tissue. Double-positive CD163+/CD55+ cells were distributed in small groups throughout proliferative areas and were significantly higher in dTGCTs (in some areas, up to 90% of proliferating cells were detected) compared to nTGCTs. Conversely, the number of non-double-positive CD163 cells was higher in nTGCT compared to dTGCT [50].

### 3.10. TRAP

The majority of multinucleated giant cells showed positive TRAP activity [89], including in the Golgi apparatus and in lysosomes [48]. TRAP activity was predominantly negative in the mononuclear cells, but was positive in a small number, ranging from 2 to 5, that tended to surround positive multinucleated cells consistent with osteoclasts [60].

### 3.11. TNF-α

Synovial expression of TNF-α was upregulated compared to controls of either normal synovium or osteoarthritis in all but one paper, with semiquantitative immunoreactivity scores of 4.35 in TGCT and 0.96 in osteoarthritis (*p* = 0.017) [52,63,69,78]. Kozhevnikov A et al., who looked specifically at juvenile arthritis, reported no significant differences in the serum TNF-α between TGCT and oligoarthritis [70]. Staining was variable across cell types, with giant cells staining most intensely, followed by histiocytes and then fibroblastic cells [78]. Mononuclear synovial cells exhibited moderate-to-slight cytoplasmic expression of TNF-α [63]. Cao showed that TNF-α, along with IL-1β, increases the expression of cadherin-11, which helps promote the migratory ability of fibroblasts, which in turn promotes its invasive potential [52].

### 3.12. IL-1β

All of the five papers that discuss IL-1β have a supporting consensus that it is positively upregulated and expressed in TGCTs. Reverse transcription polymerase chain reaction showed that an mRNA level expression of IL-1β was statistically significantly increased (*p* ≤ 0.05) compared to controls, whether in normal synovial tissue or osteoarthritis. This was also demonstrated at a protein level (*p ≤* 0.05) when using the ratio compared to GAPDH [69,71]. IL-1β staining was variable across cell types, with giant cells staining most intensely, followed by histiocytes and, lastly, fibroblastic cells [78]. Cao showed that IL-1β, along with TNF-α, also increases the expression of cadherin-11, which helps promote the migratory ability of fibroblasts, which in turn promotes its invasive potential [52]. Xie reported that dTGCTs have a more apparently invasive phenotype than nTGCTs due to specific positive differentiated gene expression involving macrophage activation, chemokine signalling, and cell disassembly. They reported that IL-1β-positive macrophages could be activated by APOE+ fibroblasts to participate in the aggressive behaviour of dTGCTs [91]. The presence of IL-1β, a known bone-resorptive cytokine in TGCT tissue, may therefore logically contribute to bone destruction and invasion.

## 4. Discussion

Tenosynovial giant cell tumours are a group of rare and complex mesenchymal tumours that cover a spectrum of diseases encompassing both nodular and diffuse forms. TGCTs have a wide phenotypic expression; however, bone destruction is one of the most severe sequalae of aggressive disease that carries significant morbidity to a generally otherwise young and healthy population. The pathogenesis and basic science of disease progression is poorly understood, meaning there is no current global consensus on disease management. The aim of this study was to systematically review the current literature and evidence on TGCT, and, in particular, those signalling factors, mechanisms, and pathways involved in disease development.

Maintaining bone homeostasis is essential, and the balance of osteoclastic and osteoblastic activity is the underpinning feature of this. Osteoclasts are unique as they are the only true bone-resorbing cells in the human body. Osteoclasts are multinucleated cells that arise from the monocyte lineage. Osteoclasts can be derived from erythromyeloid progenitors or hematopoietic stem cells. Hematopoietic stem cells differentiate into lineage-restricted precursors and then, specifically, common macrophage/osteoclast/dendritic cell progenitors (MODP) [98]. MODPs later give rise to mature osteoclasts through interactions with the nuclear factor-kappa B (NF-κB) ligand, RANK ligand, and CSF1, which are essential for the continued differentiation of mature osteoclasts [99].

After a wide and expansive search of 51 articles (Table 1), we identified 159 unique elements that had been investigated in TGCTs to varying degrees using a variety of investigative techniques, including but not limited to DNA flow cytometry, RNA sequencing, immunohistochemistry, immunofluorescence, fluorescence in situ hybridisation, enzyme assays, and bioinformatics. This heterogeneity in experimental approaches substantially limits the comparability and synthesis of findings between studies. Each technique analyses different aspects, such as protein expression, gene rearrangements, transcript/mRNA abundance, and protein quantification. This, therefore, adds inherent variability in sensitivity, specificity, and difficulty in direct comparison. Moreover, differences in sample preparation, e.g., formalin-fixed paraffin-embedded versus fresh tissue, antibody selection and scoring systems in IHC, probe design in FISH, sequencing depth and bioinformatic pipelines in RNA-seq, and calibration standards in ELISA introduce additional methodological variability. In the context of a rare condition with small patient cohorts, such inconsistencies are magnified, as limited sample sizes preclude robust stratification or cross-platform validation. Consequently, apparent discrepancies between studies may reflect methodological artefacts rather than true biological divergence, making comparisons and drawing definitive conclusions challenging. This methodological diversity, while valuable for exploratory insight, ultimately constrains the strength and generalisability of the inferences that can be drawn from the current evidence base.

A total of eight elements were reported five or more times, leading us to further analyse the results of these sub-groups. These elements included CSF1 (*n* = 14), CD68 (*n* = 11), Ki-67 (*n* = 9), MMP9 (*n* = 8), CD163 (*n* = 6), TRAP (*n* = 5), TNF-α (*n* = 5), and IL-1β (*n* = 5). Table 3 shows a cross-tabulation of each element and the relative articles. Although these elements accounted for only a small amount of the reported elements, they were noted in almost 70% of all the included articles, highlighting their prominence in the current literature.

The analysed biomarkers can be functionally categorised into four principal groups. Macrophage lineage markers include CD68, a pan-macrophage marker; CD163, indicative of alternatively activated M2 macrophages; and CSF1, a key regulator of macrophage differentiation and survival. Pro-inflammatory mediators comprise TNF-α and IL-1β, which are both central to the amplification of inflammatory responses. Markers of tissue remodelling and matrix degradation include MMP9, which facilitates extracellular matrix breakdown, and TRAP, which is associated with osteoclastic activity and tissue resorption. Finally, cellular proliferative activity is represented by Ki-67, a nuclear antigen expressed during active phases of the cell cycle. Together, these markers capture key dimensions of immune activation, inflammatory signalling, tissue remodelling, and cellular proliferation, which collectively play an intertwining role in TGCT development.

CSF1 was the most common factor to be reported in our review; this was expected as it is well established that overexpression of CSF1 due to chromosomal translocations involving the *CSF1* gene on chromosome 1p13 have been reportedly present in the majority of TGCT cases [20]. PU1 regulates the expression of *CSF1* and *RANK* gene expression in myeloid progenitor cells, resulting in the establishment of osteoclast-specific transcriptional pattern driven by RANK signalling [100]. Yoshida et al. state that mononuclear cells mediate the differentiation of TRAP-positive osteoclasts via an autocrine mechanism that involves CSF1 and RANKL [93]. CSF1 upregulation has been shown to correlate with statistically significant increases at an mRNA and protein level in TGCT compared to not only ‘healthy normal’ controls but also compared to a panel of over 200 solid tumours [64]. Therefore, increased CSF1 activity corresponds with increased osteoclast differentiation and, in turn, disrupts the bone homeostasis, resulting in increased osteoclastic activity, which, in TGCTs, can result in bone destruction

CSF1 has been a systemic target for patients with surgically unresectable disease, with CSF1 receptor inhibitors such as Pexidartinib and Vimseltinib. Following the ENLIVEN trial, the United States Food and Drug Administration approved Pexidartinib in August 2019 in the United States [101]. Currently, in the United Kingdom, there are no licenced systemic therapies for TGCT, although some therapies are used as part of a trial or off-label [16]. The National Institute for Health and Care Excellence (NICE) is currently evaluating the role of Vimseltinib in dTGCT, following the clinical trial MOTION study [102]. The MOTION trial was a randomised, placebo-controlled, double-blind study that investigated the use of Vimseltinib in patients with symptomatic dTGCTs, where surgical resection was deemed to have likely caused worsening function or severe morbidity [102,103]. There have been several clinical trials looking at systemic therapy for TGCTs, as summarised in Table 4. Imatinib is another Tyrosine Kinase Inhibitor, and was the first CSF1 receptor antagonist explored in the treatment of dTGCTs, although not as part of a clinical trial, and had mixed efficiency reported [104,105]. Identification of other therapeutic targets is essential in the development of systemic therapy for TGCTs, which remains a crucial treatment option for those with refractory or recurrent disease not amenable to surgical resection, which remains the gold-standard treatment.

Although COL6A3 was a known fusion element and was the highest single-reported fusion element in our study, the evidence suggests there is a variety of other fusion genes that can be implicated in TGCTs, including FN1, PDPN, EBF1, CD101, PRG4, TNC, and CDH17. These translocation elements can be grouped into three main groups of extracellular matrix adhesion modulators (FN1, TNC, PRG4, and CDH17), differentiation modulators (PDPN, EBF1), and immune modulators, such as CD101. Table 5 summarises the role of these elements and their interaction and resultant effects on osteoclasts. These genes integrate into a signalling network, converging on osteoclast differentiation and activation. Extracellular matrix components, such as FN1, promote osteoclast precursor adhesion via αvβ3 integrin binding, activating FAK and MAPK pathways that synergize with RANK signalling [110,111]. The increased osteoclast effect and loss of osteoclast inhibition, result in dysregulation of bone homeostasis, resulting in bone destruction in this pathological disease.

TGCT can mimic inflammatory arthropathies such as rheumatoid arthritis, with both pathologies characterised by the presence of macrophages and the expression of pro-inflammatory cytokines such as TNF-α. Anti-TNF drugs are used widely for inflammatory arthropathies, yet are not licenced for use in TGCTs. TNF-α promotes the differentiation and activation of osteoclasts and stimulates synovial cells to secrete MMPs [112]. Although our results show that synovial expression of TNF-α was upregulated compared to controls of either normal synovium or osteoarthritis in all papers involving TNF-α, the only paper to compare TGCTs to juvenile oligoarthritis reported no significant differences in the serum TNF-α [70]. Despite this, evidence suggests that cytokines that are involved in the inflammatory process in juvenile arthritis and adult rheumatoid arthritis (including TNF-α) are similar [113]. Osteoclasts are the essential link between synovial inflammation and bone destruction. TNF-α promotes the differentiation and activation of osteoclasts and, therefore, the TNF-dependent bone erosion [114].

We have already discussed the role that extracellular matrix and adhesion modulators play in osteoclast activity; the studies included in our analysis show important connections between elements in similar functional categories, such as MMP9. MMP9 expression is stimulated by TNF-α and IL-1β, which have been shown to cause periarticular bone and cartilage destruction in inflammatory conditions [78]. MMP9 expression, particularly in giant cells and histiocytes, plays a key role in matrix degradation and invasive behaviour, and is potentially enhanced by TGFβ. Therefore, this is a potential key target for TGCTs, given that bone destruction often results in end-stage disease and has not been investigated as a therapeutic target to date. TNF-α and IL-1β are soluble pro-inflammatory cytokines that can promote osteoclast differentiation via NFκB. In review of the studies included, they were consistently upregulated and may promote fibroblast migration, invasiveness, and bone resorption, especially in diffuse TGCTs.

Marked infiltration of CD68-positive macrophages was a defining histological feature across studies, reinforcing the importance of monocyte–macrophage lineage cells in TGCTs. CD68 is often involved in mediating macrophage recruitment, activation, and phagocytosis. CD68 is not exclusive to macrophages, as it can also be expressed in fibroblasts, endothelial cells, and some tumour cells. CSF1 has been shown to stimulate CD68 expression, and in vivo studies have shown that knockout of CD68 in mouse models shows that osteoclasts accumulate in intracellular vesicle-like structures, and they do not efficiently resorb bone [115]. Therefore, CD68 may not just be a marker of macrophage activity but may be a potential therapeutic target in cases of destructive bone disease.

CD163 is expressed exclusively on monocytes and macrophages, and is regarded as a marker for alternatively activated M2-type macrophages. CD163 also serves as a decoy receptor for tumour necrosis factor-like weak inducer of apoptosis (TWEAK) [116]. TWEAK is mainly expressed by macrophages/monocytes under inflammatory conditions. TWEAK has been reported to promote RANKL expression in synovial fibroblasts, which promotes bone resorption through osteoclast activity [117]. We found a macrophage-dominant phenotype, with higher double-positive cell populations in dTGCTs, suggesting a link with more aggressive disease. In vivo studies have shown that TWEAK derived from CD163- and CD68-positive macrophages was responsible for promoting bone loss, and TWEAK/Fn14 synergistically promoted RANK ligand-dependent osteoclast differentiation and mediated bone resorption [118].

Ki-67 is a marker of proliferation and active cell cycling. Analysis of the studies showed that positive Ki-67 proliferative activity was seen in mononuclear synovial cells but not in multinucleated giant cells. There was also higher expression in TGCTs than in controls. Elevated Ki-67 levels indicate rapid tumour cell division and increased osteoclast activation, which accelerates bone matrix degradation. A significant association between elevated Ki-67 levels and bone destruction has been proven in other diseases such as cholesteatoma [119]. It has been shown that Ki-67 expression is significantly higher in pathological tissues compared with normal tissue [120]. Various studies have indicated that the strategy of inhibiting Ki-67 holds promise for cancer therapies [121] but has not been investigated or trialled in TGCT to date; however, given its proven link with bone destruction, it may be an important future avenue.

TRAP is an enzyme highly expressed by osteoclasts, macrophages, and dendritic cells, and it acts as a fundamental marker for osteoclast differentiation and activity [122]. It is essential for bone remodelling, degrading bone matrix proteins (e.g., osteopontin), and facilitating mineral dissolution. The RANK ligand has been previously discussed, and TRAP activity is linked to the RANK pathway; studies show that in the presence of RANK inhibition, serum TRAP levels reflect this inhibition of osteoclast formation. In vivo studies showed that upon the return of RANK signalling, there is a rise in TRAP positivity and return of enhanced bone resorption [123]. The studies analysed show that TRAP+ mononuclear cells are precursors to bone-resorbing osteoclasts and, by surrounding osteoclasts, will contribute to lesion expansion. Therefore, TRAP positivity in multinucleated giant cells can be used as a validated indicator for osteoclast-like activity in future studies.

A key limitation of this systematic review is the rarity of the condition under investigation, which has resulted in a very limited evidence base composed predominantly of small case series and cohort numbers. The small patient numbers across included studies limit and prevent the ability to perform meaningful sub-group analyses. Furthermore, the marked heterogeneity in aetiology and pathogenesis introduces significant clinical and methodological variability, making direct comparison between studies challenging and reducing the validity of pooled estimates. Publication bias is also a concern, as rare and unusual presentations may be more likely to be reported. Collectively, these factors restrict the generalisability of findings and underscore the need for larger studies with standardised reporting frameworks.

Our study highlights the vast molecular landscape of cellular interactions involved with TGCTs, other than merely CSF1. There are many key members involved in osteoclast differentiation and activation that may be potential targets to prevent bone destruction. Although there are common principal groups of interest, there are still many potential untapped and under-investigated avenues that require further research and further meta-analyses of the current literature.

## 5. Conclusions

Apart from the well-known osteoclastogenesis factor CSF1, inflammatory cytokines TNF-α, IL-1β, and monocyte–macrophage lineage makers CD68, CD163, extracellular matrix adhesion modulators FN1, TNC, PRG4, CDH17, differentiation modulators PDPN, EBF1, and immune modulators are all involved in osteoclast activity, which plays a fundamental role in TGCT disease progression and the associated bone destruction. TGCTs are driven by a complex web of interactions at genetic, cellular, and protein levels, resulting in a difficult disease course to define and map. This molecular landscape potentially offers a wide range of potential diagnostic and therapeutic targets, but similarly, such variation may result in limited success. Further work is needed to better define and link the involved pathways to form a more cohesive understanding.

## Figures and Tables

**Table 1:** Summary of papers included in review, including patient demographics, a summary of each study, scientific techniques used, and main elements investigated.

N°	Authors	Year	Patient Numbers	Gender	Age	Main Techniques Used	Main Element Investigated
**1**	Abdul-Karim FW et al. [47]	1992	7 dTGCT 11 nTGTC 7 PVNS	dTGCT 4f/3M nTGCT 9F/2M PVNS 7F/0M	dTGCT 10–69 (median 50) nTGCT 18–71 (median 45) PVNS 19–33 (median 25)	DNA Flow Cytometry	DNA
**2**	Anazawa U et al. [48]	2006	4 GCCTS 3 PVNS 4 GCTB	Not Reported	Not Reported	Ultrastructural cytochemistry	TRAP
**3**	Berger I et al. [49]	2005	nTGCT 23 dTGCT 57	53F/27M	14–74	Immunohistochemistry DNA flow cytometry	bcl-2, p53 and Ki-67
**4**	Berger I et al. [50]	2004	nTGCT 15 dTGCT 15	Not Reported	Not Reported	Immunohistochemistry Immunofluorescence DNA flow cytometry.	CD3-, CD4-, CD8-, CD20-, CD57-, CD55-, CD68-, CD163- and h4Ph+ cells.
**5**	Berger I et al. [51]	2005	nTGCT 15 dTGCT 15	18F/12M	14–74 (mean 38)	Immunohistochemistry Immunofluorescence DNA flow cytometry	CD68, h4Ph, Ki67, CD55 and CD163
**6**	Cao C et al. [52]	2020	27	17F/10M	38.6 ± 11.3	Immunofluorescence Cell transfection	Cadherin-11, Ki-67, CD163
			4/27	Not Reported	Not Reported	Synovial fluid cytokine chip detection	RayBio^®^ Human Inflammation Antibody Array G-Series 3 AAH-INFG3 (RayBio, Ray-Biotech, USA) 45 inflammatory factors including IL-1β, TNF-α, IL-16, IL-17, IL-10, NF-κB, MAPK, COL2A1, SOX9, PI3K-Akt
**7**	Cao C et al. [53]	2021	21	13F/8M	41.7 ± 9.2	mRNA-seq array analysis Cell transfection Immunoassays	Arrb2, Ki-67, CD163, TGF-β, Rap 1, PPAR and PI3K-Akt
**8**	Chandler A C et al. [54]	2022	13	Not Reported	Not Reported	Immunocytochemistry Immunofluorescent staining DNA flow cytometry	CD90, PDPN, desmin, CSF-1, and CD14
**9**	Chen C et al. [55]	2023	3	Not Reported	Not Reported	Microarray analysis scRNA-seq	MMP9, SPP1, TYROBP
**10**	Chiang E-R et al. [56]	2015	3	Not Reported	Not Reported	DNA flow cytometry Immunohistochemical Staining Gene-expression analysis by microarrays	CD29, CD44, CD73, CD90, CD105, CD166, CD34, CD45, and CD133 COL2A1
**11**	Cupp J S et al. [57]	2007	nTGCT 31 dTGCT 36	F21/M10 F21/M15	42 mean 36 mean	In situ hybridization Immunohistochemistry	CSF1 and CSF1R
**12**	Dahlén A et al. [58]	2001	3	F3/M0	26–57 (mean 43, median 46)	Fluorescence in situ hybridization	Chromosome 7
**13**	Darling J M et al. [59]	1994	10	F6/M4	22–46 (mean 32)	In situ hybridization Immunohistochemistry	metalloproteinases, collagenase and stromelysin (MMP3)
**14**	Darling J M et al. [60]	1997	8 (6 PVNS 2 GCTTS)	Not Reported	Not Reported	In situ hybridization Immunohistochemistry	TRAP, aVI33 integrin, CD14, CTR, hCALr, HLA-DR
**15**	Ding Z et al. [61]	2021	23 nTGCT 40 dTGCT	34F/29M	18–78 (34)	Immunohistochemistry	cIAP2 and PCNA
**16**	Elkhamisy F A A et al. [62]	2024	TGCT 27	19F/8M	16–54 (mean 30.56)	Immunohistochemistry	PPARγ and P53
**17**	Finis K et al. [63]	2006	5 nTGCT 23 6 dTGCT 57			RNA labelling and hybridization Immunohistochemistry	ADAM9, ALOX5AP, ATP6V1B2, CAPG, CCL18, CCL2, CCL8, CD53, CHI3L1, CTSL, CXCR4, FRAT1, HSPA8, HSPCA, HSPCB, IFI30, LAPTM5, MAF, MAFB, MMP13, MMP9, MOAP1, MPP1, SPP1, VAMP8, ABLIM1, CXCL14, DDR2, FABP4, FHL1, KLF4, MIG-6, PLIN, S100B, CD44, TNF, TP53
**18**	Gauduchon T et al. [64]	2022	41 (57% dTGCT 43% nTGTCT)	M:F 1.4	9–74 (36.9)	RNA sequencing	CSF1, FN1, CD101, COL6A3, PRG4, TNC and CDH17
**19**	Geldyyev A et al. [65]	2007	19 nTGCT 29 dTGCT	F30/M18	18–70 (40)	RNA isolation and amplification Immunohistochemistry	RANKL, OPN, OPG, BSP, osteocalcin and PTHR
**20**	Ge M et al. [66]	2025	10 TGCT	F8/M2	36.10 ± 11.26	Immunohistochemistry	TNFSF11, CTSK, ADGRE5, NF-κB, and β-Tubulin
**21**	Ho J et al. [67]	2020	13 nTGCT 21 dTGCT 5 unclear n/dTGCT	F25/M14	21–70 (median 47)	FISH	CSF1
**22**	Ijiri K et al. [68]	2005	6 TGCT	Not Reported	Not Reported	DNA sequencing Immunohistochemistry	Humanin Peptide
**23**	Kong L et al. [69]	2025	22 TGCT	Not Reported	Not Reported	Immunohistochemical Immunofluorescence Staining DNA flow cytometry	CD90, CD206, CXCR2, MMP3, MMP9, CCL20, ACP4, M-CSF, IL-1B, and TNF-A
**24**	Kozhevnikov A et al. [70]	2025	12	F8/4M	12–16 (14.2 ± 2.6)	Enzyme immunoassay	TNF-α, and IL-6
**25**	Li T et al. [71]	2024	32	Not Reported	Not Reported	RNA-seq Immunohistochemistry Proteomics	IL-1b, TGF-b, and IL-6, CD16, CD68, MMP9, SPP1, EFEMP1, and ADAMTS18, KLF4
**26**	Mahendra G et al. [72]	2010	20	Not Reported	13–54	Immunohistochemistry	(Ki-67), apoptosis (bcl2), macrophage (CD14, CD68, HLA-DR) and osteoclast (CD51) antigens.
**27**	Molena B et al. [73]	2011	5	F1/M4	20–35	PCR and mRNA expression	CSF1
**28**	Möller E et al. [74]	2008	6	Not Reported	Not Reported	PCR	COL6A3-CSF1 Fusion
**29**	Nakashima M et al. [75]	1996	5	Not Reported	Not Reported	Immunohistochemistry In situ hybridization	PTHrP
**30**	Söder S et al. [76]	2016	10	Not Reported	Not Reported	Immunohistochemistry	CD3, CD4, CD8, CD20, CD34, CD68, CD138, mast cell tryptase (MCT), Ki-67 and CSF-1
**31**	Nilsson M et al. [77]	2002	15 dTGCT 11nTGCT	F17/M9	13–88 (mean 46)	Cytogenics FISH	DNA
**32**	O’Keefe R J et al. [78]	1998	8	F6/M2	19–80 (mean 44)	Immunohistochemistry	IL-1beta, IL-6, and TNFa. MMP-9
**33**	Ota T et al. [79]	2015	40	F26/M14	8–63 (mean 35)	Immunohistochemistry	CSF1, CSF1R, and RANKL
**34**	Seethala R R et al. [80]	2004	24	Not Reported	Not Reported	Immunohistochemistry	Microphthalmia-associated transcription factor (MITF)
**35**	Somerhausen N S [81]	2000	50	F28/M22	4–76 (median 41)	Immunohistochemistry	CD 68, Desmin, HHF35
**36**	Tang F et al. [82]	2021	4 nTGCT 2 dTGCT	F5/M1	17–67 (mean 38.8)	FISH Immunohistochemistry	CSF1R, CD163, CD68, c-Kit
**37**	Taylor R et al. [83]	2011	4	F2/M2	Not Reported	Immunohistochemistry	CD68, CD14, HLA-DR, CD51.
**38**	Thangaiah J J et al. [84]	2021	13 nTGCT 11 dTGCT 7 mTGCT	F8/M5 F4/M7 F4/M4	9–74 (47) 13–75 (38) 26–66 (57)	RNAscope CISH	CSF1
**39**	Thongchot S et al. [85]	2023	4	F1/M3	38–70 (mean 48)	Immunofluorescence Flow Cytometry Genetic Analysis	CK-19, α-SMA, FAP, PanCK and CSF1R
**40**	Uchibori M et al. [86]	2004	2 nTGCT 8 dTGCT	F5/M5	16–67 (mean 38)	Immunohistochemistry ELISA	MMP-1, -2, -3, -8, -9, and -13 and TIMP-1, -2, -3, and -4
**41**	van IJzendoorn D G P et al. [87]	2022	3 dTGCT	Not Reported	Not Reported	Immunohistochemistry Immunofluorescence FISH	CSF1, CD68, CD55, CLU, TREM1, COL6A3, POSTN, FGF7, PODN, RARRES1, CTHRC1, SLIT3, LAMB1, and ADAM12. GFPT2
**42**	Vougiouklakis T et al. [88]	2019	6	F4/M2	14–69 (mean 44)	Immunohistochemistry FISH	Non-CSF1 Fusions CD68+, CD163+ and Ki-67
**43**	Wang D D et al. [89]	2020	24	F:M 1.7:1	24–62 (mean 45.5)	Immunohistochemistry	MMP-9 MMP-13, TRAP and Ki-67
**44**	Weckauf H et al. [90]	2004	79	F53/M26	14–74 (mean 38)	Immunohistochemistry	p16, CDK4, cycline D1, RB, E2F, p53, p63, p21, p27, and Ki-67
**45**	Xie Y et al. [91]	2025	10 dTGCT 7 nTGCT	Not Reported	Not Reported	scRNA-seq Immunohistochemistry	CD34, MMP3, APOE, IL-1B, CCL20, MARCO, GFPT2, CXCL12/CXCR4, ROR1 and PRKD1, COL6A3
**46**	Yamagishi T et al. [92]	2019	9 nTGCT 10 dTGCT	F5/M4 F8/M2	Not Reported	PCR Immunohistochemistry	RANKL
**47**	Yoshida W et al. [93]	2003	10	Not Reported	Not Reported	Immunohistochemistry Enzymehistochemistry Immunofluorescence In situ hybridization	CD68, M-CSF, MIB-1, p53, p21, p16, and cath L, TRAP, MMP-2, MMP-9, RANKL, and CTR
**48**	Yudoh K et al. [94]	1999	9	F5/M4	38–71 (mean 52)	Immunohistochemistry	Telomerase Assay
**49**	Zenginkinet T et al. [95]	2022	7 nTGCT 13 dTGCT	F15/M5	14–70 (mean 40)	Immunohistochemistry	PDL-1
**50**	Zhang J et al. [96]	2022	11	Not Reported	Not Reported	Bioinformatics	DEG
**51**	Zhao Y et al. [97]	2021	6 dTGCT	Not Reported	Not Reported	RNA sequencing DNA flow cytometry Immunohistochemistry	CD3, CD6, CSF2R, CSF3R, RANK, CD45, CD56, MMP9, MMP11

**Table 2:** Risk of bias summary using the Mixed Methods Appraisal Tool, answering the questions below as either Yes (low risk of bias), Can’t tell (CT) (moderate risk), No (high risk of bias), or Not Applicable (N/A). S1. Are there clear research questions? S2. Do the collected data allow to address the research questions? 3.1. Are the participants representative of the target population? 3.2. Are measurements appropriate regarding both the outcome and intervention (or exposure)? 3.3. Are there complete outcome data? 3.4. Are the confounders accounted for in the design and analysis? 3.5. During the study period, is the intervention administered (or exposure occurred) as intended? 4.1. Is the sampling strategy relevant to address the research question? 4.2. Is the sample representative of the target population? 4.3. Are the measurements appropriate? 4.4. Is the risk of nonresponse bias low? 4.5. Is the statistical analysis appropriate to answer the research question?

**Quantitative Non-Randomised**								
		**S1**	**S2**	**3.1**	**3.2**	**3.3**	**3.4**	**3.5**
**5**	Berger I et al. [51]	Yes	Yes	Yes	Yes	Yes	N/A	Yes
**6**	Cao C et al. [52]	Yes	Yes	Yes	Yes	Yes	Yes	Yes
**7**	Cao C et al. [53]	Yes	Yes	CT	Yes	Yes	No	Yes
**8**	Chandler A C et al. [54]	Yes	Yes	CT	Yes	Yes	No	Yes
**9**	Chen C et al. [55]	Yes	Yes	CT	Yes	Yes	No	Yes
**10**	Chiang E-R et al. [56]	Yes	Yes	CT	Yes	Yes	Yes	Yes
**11**	Cupp J S et al. [57]	Yes	Yes	Yes	Yes	Yes	Yes	Yes
**12**	Dahlén A et al. [58]	Yes	Yes	No	Yes	Yes	No	Yes
**14**	Darling J M et al. [60]	Yes	Yes	CT	Yes	Yes	Yes	Yes
**17**	Finis K et al. [63]	Yes	Yes	Yes	Yes	Yes	Yes	Yes
**19**	Geldyyev A et al. [65]	Yes	Yes	Yes	Yes	Yes	Yes	Yes
**22**	Ijiri K et al. [68]	Yes	Yes	CT	Yes	Yes	Yes	Yes
**23**	Kong L et al. [69]	Yes	Yes	Yes	Yes	Yes	Yes	Yes
**24**	Kozhevnikov A et al. [70]	Yes	Yes	Yes	Yes	Yes	Yes	Yes
**25**	Li T et al. [71]	Yes	Yes	Yes	Yes	Yes	Yes	Yes
**26**	Mahendra G et al. [72]	Yes	Yes	Yes	Yes	Yes	Yes	Yes
**27**	Molena B et al. [73]	Yes	Yes	Yes	Yes	Yes	Yes	Yes
**30**	Söder S et al. [76]	Yes	Yes	Yes	Yes	Yes	Yes	Yes
**34**	Seethala R R et al. [80]	Yes	Yes	Yes	Yes	Yes	CT	Yes
**36**	Tang F et al. [82]	Yes	Yes	Yes	Yes	Yes	No	Yes
**37**	Taylor R et al. [83]	Yes	Yes	Yes	Yes	Yes	Yes	Yes
**38**	Thangaiah J J et al. [84]	Yes	Yes	Yes	Yes	Yes	Yes	Yes
**39**	Thongchot S et al. [85]	Yes	Yes	Yes	Yes	Yes	Yes	Yes
**40**	Uchibori M et al. [86]	Yes	Yes	Yes	Yes	Yes	CT	Yes
**41**	van IJzendoorn D G P et al. [87]	Yes	Yes	Yes	Yes	Yes	No	Yes
**46**	Yamagishi T et al. [92]	Yes	Yes	Yes	Yes	Yes	Yes	Yes
**48**	Yudoh K et al. [94]	Yes	Yes	Yes	Yes	Yes	Yes	Yes
**50**	Zhang J et al. [96]	Yes	Yes	Yes	Yes	Yes	Yes	Yes
**51**	Zhao Y et al. [97]	Yes	Yes	CT	Yes	Yes	Yes	Yes
**Quantitative Descriptive**								
		**S1**	**S2**	**4.1**	**4.2**	**4.3**	**4.4**	**4.5**
**1**	Abdul-Karim FW et al. [47]	Yes	Yes	CT	CT	Yes	N/A	Yes
**2**	Anazawa U et al. [48]	Yes	Yes	CT	CT	Yes	N/A	Yes
**3**	Berger I et al. [49]	Yes	Yes	Yes	Yes	Yes	N/A	Yes
**4**	Berger I et al. [50]	Yes	Yes	CT	Yes	Yes	N/A	Yes
**13**	Darling J M et al. [59]	Yes	Yes	CT	Yes	CT	No	CT
**15**	Ding Z et al. [61]	Yes	Yes	Yes	Yes	Yes	N/A	Yes
**16**	Elkhamisy F A A et al. [62]	Yes	Yes	Yes	Yes	Yes	N/A	Yes
**18**	Gauduchon T et al. [64]	Yes	Yes	Yes	Yes	Yes	N/A	Yes
**20**	Ge M et al. [66]	Yes	Yes	Yes	Yes	Yes	N/A	Yes
**21**	Ho J et al. [67]	Yes	Yes	CT	No	Yes	N/A	Yes
**28**	Möller E et al. [74]	Yes	Yes	CT	CT	Yes	N/A	Yes
**29**	Nakashima M et al. [75]	Yes	Yes	CT	Yes	Yes	N/A	Yes
**31**	Nilsson M et al. [77]	Yes	Yes	CT	Yes	Yes	N/A	Yes
**32**	O’Keefe R J et al. [78]	Yes	Yes	CT	No	Yes	N/A	Yes
**33**	Ota T et al. [79]	Yes	Yes	Yes	Yes	Yes	N/A	Yes
**35**	Somerhause N S [81]	Yes	Yes	Yes	Yes	Yes	N/A	Yes
**42**	Vougiouklakis T et al. [88]	Yes	Yes	No	No	Yes	N/A	Yes
**43**	Wang D D et al. [89]	Yes	Yes	CT	Yes	Yes	N/A	Yes
**44**	Weckauf H et al. [90]	Yes	Yes	Yes	Yes	Yes	N/A	Yes
**45**	Xie Y et al. [91]	Yes	Yes	CT	Yes	Yes	N/A	Yes
**47**	Yoshida W et al. [93]	Yes	Yes	CT	Yes	Yes	N/A	Yes
**49**	Zenginkinet T et al. [95]	Yes	Yes	Yes	Yes	Yes	N/A	Yes

**Table 3:** Cross-tabulation of the factors reported five or more times with the articles they are reported in.

N°	Authors	CSF1	CD68	Ki-67	MMP9	CD163	TRAP	TNF-α	IL-1β
**2**	Anazawa U et al. [48]						X		
**3**	Berger I et al. [49]			X					
**4**	Berger I et al. [50]		X			X			
**5**	Berger I et al. [51]		X	X		X			
**6**	Cao C et al. [52]			X		X		X	X
**7**	Cao C et al. [27]			X		X			
**8**	Chandler A C et al. [54]	X							
**9**	Chen C et al. [55]				X				
**11**	Cupp J S et al. [57]	X							
**14**	Darling J M et al. [60]						X		
**17**	Finis K et al. [63]							X	
**18**	Gauduchon T et al. [64]	X							
**21**	Ho J et al. [67]	X							
**23**	Kong L et al. [69]	X			X			X	X
**24**	Kozhevnikov A et al. [70]							X	
**25**	Li T et al. [71]		X		X				X
**26**	Mahendra G et al. [72]		X	X					
**27**	Molena B et al. [73]	X							
**28**	Möller E et al. [74]	X							
**30**	Söder S et al. [76]	X	X	X					
**32**	O’Keefe R J et al. [78]				X			X	X
**33**	Ota T et al. [79]	X					X		
**35**	Somerhausen, N S [81]		X						
**36**	Tang F et al. [82]	X	X			X			
**37**	Taylor R et al. [83]		X						
**38**	Thangaiah J J et al. [84]	X							
**39**	Thongchot S et al. [85]	X							
**40**	Uchibori M et al. [86]				X				
**41**	van IJzendoorn D G P et al. [87]	X	X						
**42**	Vougiouklakis T et al. [88]		X	X		X			
**43**	Wang D D et al. [89]			X	X		X		
**44**	Weckauf H et al. [90]			X					
**45**	Xie Y et al. [91]								X
**47**	Yoshida W et al. [93]	X	X		X		X		
**51**	Zhao Y et al. [97]				X				

**Table 4:** Summary of clinical trials for TGCTs, drugs used, and their mechanism of action.

Trial Name	Drug	Mechanism of Action
MOTION [102,103]	Vimseltinib	CSF1 receptor inhibitor
MANEUVER [106]	Pimicotinib	CSF1 receptor inhibitor
ENLIVEN [101]	Pexidartinib	CSF1 receptor inhibitor
TANGENT [107]	Emactuzumab	CSF1 receptor monoclonal antibody
AMB-05X [108]	AMB-05X	CSF1 receptor monoclonal antibody
Cabiralizumab [109]	Cabiralizumab	CSF1 receptor monoclonal antibody

**Table 5:** Summary of translocation elements identified and their interaction and resultant effect on osteoclast activity.

Gene	Product	Role	Interaction with Osteoclasts	Effect
*FN1*	Glycoprotein	Adhesion, migration and differentiation	Binds αvβ3 integrin and promotes osteoclast adhesion	Enhances osteoclasts
*PDPN*	Glycoprotein	Remodelling and cell motility	Modulates RhoA signalling and osteoclast precursor migration	Indirect promoter of osteoclast formation
*EBF1*	Transcription factor	Regulator of cell lineage differentiation	Indirectly affects RANK ligand balance	Influences osteoclast differentiation
*CD101*	Immunoglobulin receptor	Immune modulation	Increase inflammatory cytokine production	Modulate osteoclast differentiation
*PRG4*	Glycoprotein	Cell proliferation	Downregulation promotes RANKL expression	Loss of osteoclast protection
*TNC*	Glycoprotein	Remodelling and inflammation	Binds integrins and modulates adhesion, enhancing RANKL	Influences osteoclast differentiation
*CDH17*	Calcium-dependent adhesion molecule	Adhesion	Influence osteoclast adhesion	Enhances osteoclasts

## Data Availability

No new data were created or analyzed in this study.

## Supplementary Materials

The following supporting information can be downloaded at: https://www.mdpi.com/article/10.3390/jcm15062238/s1.

## Abbreviations

The following abbreviations are used in this manuscript:

TGCT	Tenosynovial giant cell tumour
nTGCT	Nodular tenosynovial giant cell tumour
dTGCT	Diffuse tenosynovial giant cell tumour
UK	United Kingdom
PVNS	Pigmented villonodular synovitis
WHO	World Health Organisation
M-CSF	Macrophage colony-stimulating factor
CSF1	Colony-stimulating factor 1
RANK	Receptor activator of nuclear factor kappa-Β
DNA	Deoxyribose nucleic acid
CD	Cluster of differentiation
TRAP	Tartrate-resistant acid phosphatase
Ki-67	Antigen Kiel 67
MMP	Matrix metalloproteinase
TNF-α	Tumour necrosis factor alpha
IL-1β	Interleukin-1 beta
FN1	Fibronectin 1
PDPN	Podoplanin
EBF1	Early B-cell factor 1
PRG4	Proteoglycan 4
TNC	Tenascin-C
CDH17	Cadherin-17
GAPDH	Glyceraldehyde 3-phosphate dehydrogenase

## Appendix A

The following search terms were used to conduct the literature searches.

Scopus: (TITLE-ABS-KEY (Tenosynovial Giant Cell Tumour*) OR TITLE-ABS-KEY (TGCT) OR TITLE-ABS-KEY (PVNS) OR TITLE-ABS-KEY (Pigmented Villonodular Synovitis) AND TITLE-ABS-KEY (pathways) OR TITLE-ABS-KEY (signalling) OR TITLE-ABS-KEY (molecular) OR TITLE-ABS-KEY (mechanism) OR TITLE-ABS-KEY (bone destruction) OR TITLE-ABS-KEY (analysis) OR TITLE-ABS-KEY (osteoclast) AND NOT TITLE-ABS-KEY (Testicular*) OR TITLE-ABS-KEY (Case Report)).

PubMed: (((((Tenosynovial giant cell tumour[Title/Abstract]) OR (TGCT[Title/Abstract])) OR (PVNS[Title/Abstract])) OR (Pigmented Villonodular synovitis[Title/Abstract])) AND (pathways[Title/Abstract] OR signalling[Title/Abstract] OR molecular[Title/Abstract] OR mechanism[Title/Abstract] OR bone destruction[Title/Abstract] OR analysis[Title/Abstract] OR Osteoclast[Title/Abstract])) NOT (Testicular[Title/Abstract] OR Case Report[Title/Abstract]).

Cochrane Database: (Tenosynovial Giant Cell Tumour* OR TGCT OR PVNS OR Pigmented Villonodular Synovitis) AND (pathways OR signalling OR molecular OR mechanism OR bone destruction OR analysis OR Osteoclast) NOT (Testicular OR ‘Case Report’).
